# AI-Assisted Diagnostic Evaluation of IHC in Forensic Pathology: A Comparative Study with Human Scoring

**DOI:** 10.3390/diagnostics16010006

**Published:** 2025-12-19

**Authors:** Francesco Sessa, Mara Ragusa, Massimiliano Esposito, Mario Chisari, Cristoforo Pomara, Monica Salerno

**Affiliations:** 1 Department of Psychology and Health Sciences, Faculty of Human Sciences, Education, and Sports, Pegaso Telematic University, 80143 Naples, Italy; 2 Department of Medical, Surgical and Advanced Technologies “G.F. Ingrassia”, University of Catania, 95121 Catania, Italy; mara.ragusa@outlook.it (M.R.); cristoforo.pomara@unict.it (C.P.); monica.salerno@unict.it (M.S.); 3 Faculty of Medicine and Surgery, “Kore” University of Enna, 94100 Enna, Italy; massimiliano.esposito@unikore.it (M.E.); mario.chisari@unikore.it (M.C.)

**Keywords:** immunohistochemistry, artificial intelligence, forensic pathology, generative models, digital pathology, machine learning, image classification, diagnostic accuracy, explainable AI, post-mortem interval

## Abstract

**Background/Objectives**: Immunohistochemistry (IHC) is a critical diagnostic tool in forensic pathology, enabling molecular-level assessment of wound vitality, post-mortem interval, and cause of death. However, IHC interpretation is subject to variability due to its reliance on human expertise. This study investigates whether artificial intelligence (AI), specifically a generative model, can assist in the diagnostic evaluation of IHC slides and replicate expert-level scoring, thereby improving consistency and reproducibility. **Methods**: A total of 225 high-resolution IHC images were classified into five immunoreactivity categories. The AI model (ChatGPT-4V) was trained on 150 labeled images and tested blindly on 75 unseen slides. Performance was assessed using confusion matrices, per-class precision/recall/F1, overall accuracy, Cohen’s κ (unweighted and weighted), and binary metrics (sensitivity, specificity, MCC). **Results**: Overall accuracy was 81.3% (95% CI: 71.1–88.5%), with substantial agreement (κ = 0.767 unweighted; 0.805 linear-weighted; 0.848 quadratic-weighted). Binary classification achieved a sensitivity of 98.3%, specificity of 93.3%, MCC of 0.92. Accuracy was highest in extreme categories (− and +++, 93.3%), while intermediate classes (+ and ++) showed reduced performance (error rates up to 33%). Evaluation was rapid and consistent but lacked interpretative reasoning and struggled with borderline cases. **Conclusions**: AI-assisted diagnostic evaluation of IHC slides demonstrates promising accuracy and consistency, particularly in well-defined staining patterns. While not a replacement for human expertise, AI can serve as a valuable adjunct in forensic pathology, supporting rapid and standardized assessments. Ethical and legal considerations must guide its implementation in medico-legal contexts.

## 1. Introduction

Immunohistochemistry (IHC) is an essential tool in forensic pathology, providing molecular-level insights that complement traditional histology [1]. IHC visualizes specific antigens in tissue, enabling precise assessment of cellular responses, injury vitality, post-mortem interval, and cause of death [2,3,4,5]. The technique integrates principles from histology, immunology, and biochemistry, relying on monoclonal and polyclonal antibodies to detect target proteins. Through a sequence of steps, including antigen retrieval, antibody binding, and chromogenic detection, IHC provides spatial and quantitative data that are critical in forensic investigations, especially when conventional stains fail to reveal significant pathological changes [6]. Unlike conventional AI-based scoring systems that rely on deterministic convolutional neural networks (CNNs) trained for specific biomarkers, generative models such as ChatGPT-4V operate through multimodal prompt-driven interactions, enabling flexible interpretation across heterogeneous staining patterns without retraining [7].

Despite its diagnostic power, IHC interpretation is not without challenges. The accuracy of results is influenced by numerous pre-analytical, analytical, and post-analytical variables. Tissue handling, fixation delays, and staining protocols can introduce inconsistencies, while the final evaluation often depends on the subjective judgment of pathologists. This reliance on human interpretation introduces inter-observer variability, particularly in cases involving degraded or autolyzed tissues [8]. Experienced pathologists may better distinguish true immunoreactivity from artifacts, but the process remains time-consuming and prone to bias. Moreover, many of the experimental studies are performed with limited sample sizes and inconsistent population standardization. This raises concerns about the scientific validation of IHC techniques used in forensic settings [9].

Historically, IHC has evolved from a research-oriented technique to a cornerstone of diagnostic pathology, including forensic applications [10,11,12]. However, its integration into routine forensic workflows remains uneven across institutions, partly due to the lack of standardized scoring systems and reproducible protocols.

In forensic practice, the classification of immunopositivity often requires the consensus of two independent pathologists to ensure diagnostic reliability. This dual evaluation is particularly important in borderline cases, where staining intensity may be ambiguous or affected by post-mortem degradation [13,14]. However, in a forensic context, obtaining two expert opinions for every slide can be resource-intensive, especially in high-throughput settings or institutions with limited personnel. The need for reproducibility and legal defensibility further amplifies the importance of second opinions, but also increases operational costs and delays. In this context, AI-assisted evaluation offers a promising alternative. By providing a rapid, standardized, and reproducible second opinion, AI can support pathologists in making more confident decisions while optimizing time and cost [15,16]. Such systems could be particularly valuable in preliminary screening, triage, or quality control, allowing human experts to focus on complex or legally sensitive cases. The integration of AI into post-mortem IHC workflows may thus enhance diagnostic efficiency without compromising scientific rigor.

In this context, digital pathology has emerged as a transformative approach, converting traditional slides into high-resolution digital images suitable for computational analysis. Within this framework, artificial intelligence (AI), especially machine learning (ML) and deep learning (DL) techniques, has shown promise in automating image interpretation. These systems can learn from labeled datasets, recognize complex patterns, and perform classification tasks with consistency and scalability. While AI has already demonstrated utility in clinical pathology, its application in forensic contexts remains underexplored [17,18]. The application of AI tools in forensic sciences constitutes a challenging and rapidly evolving area of research that is attracting growing scholarly interest [19,20,21,22,23].

Recent advances in AI-assisted diagnostic tools have shown high accuracy in clinical IHC scoring, particularly in oncology, where DL models have achieved near-human performance [15]. These developments suggest that similar approaches could be adapted to forensic pathology, where diagnostic consistency is critical for legal outcomes.

Moreover, the potential for AI to reduce diagnostic variability, accelerate image evaluation, and support decision-making in complex forensic scenarios makes it a compelling candidate for integration into forensic workflows [16]. Recent regulatory frameworks, including FDA (Food and Drug Administration) guidance on Software as a Medical Device (SaMD) and College of American Pathologists (CAP) recommendations for AI validation in pathology, emphasize reproducibility, transparency, and explainability as prerequisites for clinical and forensic adoption.

This study aims to investigate the feasibility of integrating generative AI models into the evaluation of IHC slides in forensic pathology. By training and testing an AI system on a structured dataset of immunostained images, the research seeks to determine whether such models can replicate expert-level scoring and reduce diagnostic variability. The broader goal is to assess how AI-assisted diagnostic tools might support forensic pathologists by offering standardized, reproducible, and rapid assessments of immunoreactivity, ultimately enhancing the reliability and efficiency of forensic diagnostics.

## 2. Materials and Methods

### 2.1. Study Design and Setting

This experimental study was conducted at the Section of Forensic Medicine, University of Catania. It aimed to evaluate the performance of a generative AI model (ChatGPT-4, February 2024 update, a publicly accessible AI model developed by OpenAI, URL: https://openai.com, accessed 15 May 2025) in classifying IHC images, comparing its results with expert forensic pathologists. The study comprised three phases: image selection and classification, AI training, and blind testing on unseen images. The study protocol was designed to ensure reproducibility and transparency in each phase.

### 2.2. Ethical Approval and Data Use

All procedures performed in this study adhered to the ethical standards of the institutional research committee and the 1964 Helsinki Declaration and its later amendments or comparable ethical standards. The study received approval from the Ethics Committee of Catania Hospital (approval code: 04/CEL). Data governance adhered to GDPR and institutional policies. Images were anonymized, and no personally identifiable information was processed. The AI interaction logs were stored securely and will be made available upon request.

Under Italian regulations, written informed consent was not required, as the study involved the collection and analysis of secondary data from judicial autopsy cases. All data were anonymized prior to analysis.

### 2.3. Rationale for Laboratory Variability

Unlike conventional studies that specify antibody clones, dilutions, and antigen retrieval protocols, our experimental design intentionally incorporated heterogeneity in these parameters. The images analyzed were sourced from the archival collection of the Institute of Legal Medicine, encompassing diverse tissues, antibodies, and staining conditions. All reactions shared the common feature of a brown chromogenic signal, ensuring visual comparability while maintaining variability in technical details.

This design simulates real-world forensic conditions and evaluates AI as a reliable second opinion, independent of laboratory protocols. Both forensic pathologists and the AI system assessed the slides in a blind manner, without access to underlying laboratory information. By avoiding overfitting to a single marker or protocol, this design enhances external validity and supports the broader goal of testing AI as a generalizable adjunct tool for forensic IHC.

Although pre-analytical, analytical, and post-analytical factors can affect IHC interpretation, their impact was minimized by strict inclusion/exclusion criteria and consensus validation by two forensic pathologists. Only high-quality, diagnostically reliable images were analyzed, ensuring that variability did not compromise the accuracy of AI or human evaluations.

### 2.4. Image Source and Acquisition

Digital images were retrieved from the institutional archive of the Institute of Forensic Medicine, University of Catania. All cases had been previously evaluated between 2015 and 2018 and included a range of forensic scenarios (e.g., trauma, hypoxia, inflammation).

Tissue samples were processed using standardized IHC protocols: tissue samples were processed using conventional immunohistochemistry protocols, including formalin fixation, paraffin embedding, sectioning at 4–5 μm, antigen retrieval, incubation with primary and secondary antibodies, and chromogenic detection using DAB (diaminobenzidine) (Abcam Limited, Cambridge, UK). All slides were counterstained with hematoxylin and mounted for microscopic evaluation. Images were acquired using a Leica microscope at magnifications of 5×, 10×, 20×, and 40×, and saved in TIFF format to preserve resolution and detail. Images were used in their original TIFF format without additional preprocessing (e.g., normalization, color balancing, or contrast adjustment) to preserve diagnostic integrity and avoid introducing artifacts. All images were acquired using a Leica microscope (Leica, Wetzlar, Germany) under controlled laboratory conditions (Table S1).

To ensure the reliability and consistency of the dataset, strict inclusion and exclusion criteria were applied during image selection. Only high-resolution images that displayed clear immunohistochemical staining patterns were considered eligible for analysis. Each selected image had to be validated by two independent forensic pathologists to confirm its diagnostic quality and accurate classification.

Conversely, images were excluded if they showed poor resolution or technical artifacts such as tissue folding, tearing, overstaining, or uneven chromogen deposition. This approach was adopted to minimize interpretative uncertainty and to ensure that both human and AI evaluations were based on well-defined and reproducible visual features.

Each image was classified into one of five immunoreactivity categories based on the percentage of positively stained cells, as suggested by Meyerholz and Beck [24] (Table 1):

For clarity, the term “immunoreactivity score” refers to the ordinal category (0–4) assigned based on the percentage of immunopositive cells (Table 1). “Staining intensity” is used descriptively but corresponds to these categories. “Accuracy” denotes the proportion of correctly classified images across all five categories during evaluation.

A total of 225 images were selected and evenly distributed across the five categories (45 images per category). These were further divided into the following:

- Training set: 30 images per category (150 total).
- Blind testing set: 15 images per category (75 total).

### 2.5. AI Training Protocol

The AI model used was ChatGPT-4V (OpenAI), a proprietary generative system with multimodal capabilities. As this model is not open-source and its internal architecture is undisclosed, reproducibility is limited. To mitigate this, we documented all prompts, interaction protocols, and image preprocessing steps in Supplementary Material (Table S2). The model was accessed via a paid subscription to enable image upload and memory retention features.

The training phase consisted of structured prompt engineering rather than algorithmic fine-tuning, as ChatGPT-4 does not allow parameter-level adjustments. This approach simulates supervised learning but does not constitute true model retraining. We acknowledge this limitation and clarify that the AI’s performance reflects prompt-based adaptation rather than intrinsic model optimization. Initially, each image in the training dataset was manually labeled according to its immunoreactivity score, ranging from negative (−) to strongly positive (++++) based on the percentage of stained cells. Each of the 225 images was independently evaluated by two forensic pathologists, resulting in 450 human observations. Consensus was reached through discussion in cases of disagreement, and these consensus scores served as the ground truth for AI comparison.

To guide the AI’s learning process, the model was introduced to the scoring framework through descriptive prompts and representative examples for each category. These instructions were embedded in the interaction to simulate a supervised learning environment.

Images were then uploaded sequentially into the AI interface. For each image, the model was prompted with a standardized question:

“This image has been evaluated as [score]. What is your opinion?”

This approach allowed the AI to compare its own interpretation with the reference classification and adjust its responses accordingly.

Throughout the training, the AI’s outputs were monitored and compared to the ground truth labels. Any discrepancies or misclassifications were documented and used to reinforce the model’s understanding through repeated exposure to similar examples. Feedback consisted of prompt-based reinforcement: when the AI misclassified an image, the correct category was communicated in the next interaction (e.g., ‘Your previous classification was incorrect. The correct score is [score].’). This iterative approach allowed the model to refine its responses without parameter-level retraining. This iterative feedback loop helped the AI refine its pattern recognition and align more closely with expert-level evaluations.

### 2.6. Blind Testing Phase

To assess generalization and independent classification ability, the AI was tested on the remaining 75 images. This phase was conducted under the following conditions:

- Randomization: Images were uploaded in a randomized order to prevent pattern recognition based on sequence.
- No Label Disclosure: The AI was not informed of the expected score.
- Independent Evaluation: The AI was asked to classify each image based solely on visual features, without contextual cues.

### 2.7. Statistical Analysis

Agreement between AI and pathologist classifications was assessed using a 5 × 5 confusion matrix, overall accuracy (95% CI via Wilson method), and Cohen’s Kappa (unweighted, linear-weighted, and quadratic-weighted). Confidence intervals for κ were obtained via nonparametric bootstrap (2000 replicates). Per-class precision, recall, and F1-score were computed, alongside binary metrics (sensitivity, specificity, balanced accuracy, MCC) for negative vs. positive grouping. Misclassification severity was characterized as adjacent (±1 level) or non-adjacent (≥2 levels). Visualization of classification patterns was provided through a confusion matrix.

## 3. Results

### 3.1. Performance During the Training Phase

In the initial training phase (T0), the AI model was exposed to a structured dataset of 150 images, evenly distributed across five immunoreactivity categories. The model was prompted with labeled examples and asked to classify each image according to the predefined scoring system. The AI demonstrated a high level of adaptability, progressively aligning its responses with the human-assigned scores.

The binary classification between negative (−) and weakly positive (+) images showed near-perfect accuracy, with only two misclassifications in the “−” category and none in the “+” group. This initial performance is summarized in Table 2.

Subsequently, the model was tested across all five categories. The AI correctly classified all images in the “+”, “++”, “+++”, and “++++” groups, while maintaining a 93.3% accuracy in the “−” category. These results are detailed in Table 3, which presents the error rates and correct classification percentages for each class.

### 3.2. Blind Evaluation Phase

Following training, the model underwent blind testing (T1) using 75 previously unseen images. These were uploaded in a randomized sequence to prevent bias and simulate real-world diagnostic conditions. The AI was tasked with independently classifying each image without prior knowledge of its category.

The model maintained high accuracy in the “−” and “+++” categories, correctly classifying 93.3% of images in both groups. However, performance declined in intermediate categories, particularly “++”, where the accuracy dropped to 66.7%. These results are presented in Table 4 (binary classification, “negative” vs. “positive”) and Table 5 (full category breakdown).

**Figure 1:**
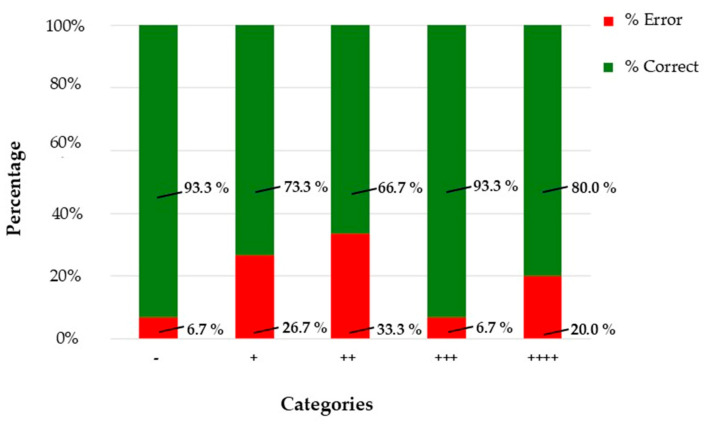
Classification results for blind testing phase (n = 75 images) across five immunoreactivity categories. Bars represent correct (green) and incorrect (red) classifications; percentages are annotated above each bar.

To visually represent these findings, Figure 1 shows the number and percentage of correct (green) and incorrect (red) classifications for each immunoreactivity category (n = 15 per class). Accuracy was highest for ‘−’ and ‘+++’ (93.3%), while intermediate categories (‘+’, ‘++’) exhibited greater variability, with error rates up to 33%.

### 3.3. Comparison with Pathologist Evaluation

**Figure 2:**
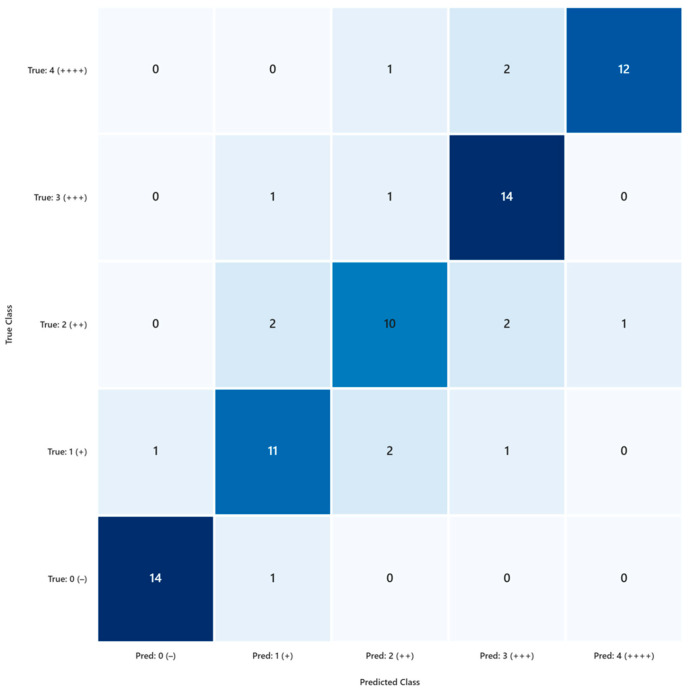
Confusion matrix comparing AI-predicted immunoreactivity scores with ground-truth pathologist labels during blind testing (n = 75 images). Diagonal cells represent correct classifications, while off-diagonal cells indicate misclassifications. The AI achieved high concordance in extreme categories (0 [−] and 3 [+++], each 93.3% correct), whereas intermediate categories (+ and ++) showed greater variability, with error rates up to 33.3%. Overall accuracy was 81.3%, and Cohen’s κ confirmed substantial agreement (unweighted κ = 0.767; linear-weighted κ = 0.805; quadratic-weighted κ = 0.848). Percentages in parentheses denote the proportion of each cell relative to the total dataset.

The agreement between AI predictions and ground-truth pathologist labels was assessed using multiple metrics. Overall accuracy reached 81.3% (61/75; 95% CI: 71.1–88.5%), confirming strong concordance across the five immunoreactivity categories. Figure 2 illustrates the confusion matrix, where diagonal cells represent correct classifications and off-diagonal entries indicate misclassifications. Most errors occurred in intermediate categories, particularly “+” (26.7% error) and “++” (33.3% error), while extreme categories such as “−” and “+++” showed high agreement (93.3% accuracy each).

Cohen’s Kappa confirmed substantial agreement:

- Unweighted κ = 0.767
- Linear-weighted κ = 0.805
- Quadratic-weighted κ = 0.848

These values indicate robust concordance even when accounting for ordinal misclassification severity. Error analysis revealed six adjacent misclassifications (±1 level) and eight non-adjacent errors, underscoring the challenge of borderline cases.

Per-class performance (Precision/Recall/F1-score):

- 0 (−): 0.93/0.93/0.93
- 1 (+): 0.92/0.73/0.81
- 2 (++): 0.91/0.67/0.77
- 3 (+++): 0.78/0.93/0.85
- 4 (++++): 0.63/0.80/0.71

Binary classification (negative vs. positive) showed excellent performance:

- Sensitivity = 98.3%
- Specificity = 93.3%
- Balanced accuracy = 95.8%
- Matthews Correlation Coefficient (MCC) = 0.92

These findings confirm that the AI model performs best in clearly defined staining categories (negative and strongly positive), while intermediate scores remain more challenging. From a practical perspective, a κ > 0.8 indicates substantial agreement, supporting the use of AI as a second-opinion tool rather than a primary diagnostic system.

In addition to accuracy, the AI demonstrated exceptional speed and consistency, completing the evaluation of 75 images in a fraction of the time required by human pathologists. However, the model lacked interpretative reasoning and contextual awareness, which remain essential in forensic diagnostics. A summary of qualitative observations is provided in Table 6, highlighting strengths (speed, reproducibility, high performance in extreme categories) and weaknesses (lower recall in intermediate classes, lack of explainability).

## 4. Discussion

Prompt-engineered generative AI approximates human-level performance under controlled conditions but does not confer diagnostic capability or regulatory compliance. The model achieved substantial agreement (κ = 0.767 unweighted; 0.805 linear-weighted; 0.848 quadratic-weighted), confirming robust concordance. However, intermediate categories (+ and ++) showed reduced performance, with error rates up to 33.3%, compared to 93.3% accuracy in extreme classes. Generative models lack domain-specific optimization and explainability, which are essential for clinical or forensic deployment [25]. The model achieved high accuracy in both the training and blind testing phases, particularly in categories with clearly defined staining patterns. These results support the hypothesis that AI can serve as a valuable adjunct in forensic diagnostics, offering consistency, speed, and scalability. Despite these positive results, a recent publication recommended its use under human supervision [26,27,28]. It is important to note that this study does not aim to validate AI for primary diagnosis or patient care as outlined in RCPath guidelines [29]; rather, its purpose is to explore AI as a supportive tool for forensic pathologists in semi-quantitative IHC scoring (positive/negative and intensity classes), addressing workforce shortages and improving consistency in non-clinical, medico-legal contexts. Bias was minimized through a dual-review process: each image was independently evaluated by two forensic pathologists, and consensus was reached in cases of disagreement. This approach reduces individual subjectivity and ensures diagnostic reliability. Additionally, AI-assisted scoring provides a standardized second opinion, further mitigating interpretative bias without replacing human judgment.

During the training phase, the AI model showed near-perfect classification accuracy across all five immunoreactivity categories. This suggests that, under controlled conditions with labeled data, the model can effectively learn and replicate the scoring criteria used by expert pathologists. In the blind evaluation phase, the model maintained strong performance in the “−” and “++++” categories, with over 93% accuracy. These categories represent the extremes of the staining spectrum, where visual features are more distinct and less prone to interpretative ambiguity. However, the model’s performance declined in intermediate categories, particularly “++”, where accuracy dropped to 66.7%. This reflects a known challenge in IHC interpretation: borderline cases often involve subtle differences in staining intensity and distribution, which are difficult to quantify even for experienced pathologists. The confusion matrix and F1-score analysis further confirmed this limitation, highlighting the need for improved sensitivity in mid-range classifications.

These findings align with previous studies in clinical pathology. Wang et al. reported that an AI tool can achieve high accuracy and strong concordance with conventional IHC, offering a promising assistive application for cancer diagnostics. In particular, DL-based IHC biomarker models have demonstrated AUCs of up to 0.96, streamlining pathology workflows while maintaining diagnostic reliability [30]. Similarly, Darbandi et al. explored the application of AI for early breast cancer detection, reviewing 310 studies and performing experimental analyses across 30 datasets. Their results showed that recurrent neural networks achieved the highest accuracy (98.58%), followed closely by genetic principles, transfer learning, and artificial neural networks, all exceeding 96% accuracy [31]. The observed accuracy reflects controlled conditions: images were pre-validated by experts, and variability was limited to real-world heterogeneity in staining protocols rather than uncontrolled artifacts. This approach supports external validity while maintaining diagnostic reliability.

In line with these results, Uwimana et al. conducted a scoping review of AI and ML applications in breast cancer clinical care, emphasizing their synergy with medical imaging in improving diagnostic accuracy, reducing false positives, and enabling personalized treatment strategies [32]. Complementing these insights, Jung et al. validated an artificial neural network-based diagnostic system for prostate biopsy interpretation, demonstrating expert-level accuracy in cancer detection and grading, and outperforming original pathology reports in concordance with reference Gleason scores and grade groups [33].

The application of AI in forensic IHC presents unique challenges and opportunities. Unlike clinical pathology, forensic cases often involve degraded or autolyzed tissues, variable fixation protocols, and complex medico-legal contexts. The markers used are critical for determining wound vitality, hypoxia, inflammation, and the cause of death. Accurate interpretation of these markers can influence legal outcomes, making consistency and reproducibility essential. Recent systematic reviews have highlighted that AI applications in forensic pathology can achieve high accuracy across diverse domains, including 87.99–98% accuracy in gunshot wound classification, precision scores of 0.9 and recall scores of 0.95 in diatom testing for drowning cases, and up to 90% accuracy in microbiome-based individual identification and geographic origin determination [19,20,34,35]. These results underscore the capacity of AI to process complex forensic evidence and support objective decision-making in legally sensitive contexts. The AI model’s ability to classify images based on these markers suggests that, with further refinement, such systems could support forensic pathologists in routine and complex cases. In line with recent evidence, AI should be considered an enhancement rather than a replacement for human expertise, with future development focusing on larger forensic datasets, specialized models for specific applications, and improved interpretability to meet legal admissibility standards. Integrating AI into these workflows could enhance diagnostic precision and reduce inter-observer variability [36,37,38].

### 4.1. Strengths and Limitations

One of the key strengths of the AI model was its speed and consistency. The system evaluated 75 images in a fraction of the time required by human pathologists, without fatigue or bias. This efficiency could be particularly valuable in high-volume forensic laboratories or in settings with limited access to specialized expertise.

However, the model also exhibited notable limitations. It lacked interpretative reasoning and contextual awareness, providing classifications without explaining the rationale behind its decisions. This “black box” nature of AI raises concerns about transparency and trust, especially in legal contexts where expert testimony must be justified and reproducible.

Moreover, the study was limited by its sample size and dataset homogeneity. All images were sourced from a single institution, potentially introducing bias related to staining protocols and image acquisition. Future studies should incorporate multi-center datasets and a broader range of tissue types and markers to improve generalizability.

Finally, this study is exploratory and does not meet RCPath criteria for staged validation or external benchmarking. Future work will incorporate multi-center datasets, independent validation, and compliance with RCPath recommendations for transparency, auditability, and medico-legal accountability.

### 4.2. Implications for Practice and Future Research

The integration of AI into forensic pathology is not merely a technical innovation; it represents a paradigm shift in how diagnostic decisions are made and validated. As previously discussed, AI offers benefits in standardization, accessibility, and cost-effectiveness. It can democratize access to advanced IHC analysis, especially in regions with limited forensic infrastructure.

To fully realize this potential, future research should focus on the following:

- Expanding training datasets to include diverse staining patterns and tissue conditions.
- Developing explainable AI models that provide visual or textual justifications for their classifications.
- Validating AI systems across multiple institutions and forensic scenarios.
- Exploring integration with other modalities, such as radiology and toxicology, to create multimodal forensic diagnostic platforms.

Moreover, considering the need for dual expert opinions in forensic IHC evaluation, our results suggest that AI systems could serve as a reliable second evaluator, complementing the human pathologist. This approach could optimize both diagnostic accuracy and operational efficiency. By integrating AI into routine workflows, institutions could reduce the time and cost associated with obtaining multiple expert reviews, especially in high-throughput or resource-limited settings. The AI model’s strong performance in clearly defined staining categories supports its use as a preliminary or confirmatory tool, helping to flag discrepancies or reinforce consensus. Importantly, this dual evaluation strategy maintains the integrity of forensic diagnostics while leveraging the scalability and reproducibility of AI. This study used a proprietary AI system (ChatGPT-4V v 3.5), which limits reproducibility due to a lack of access to model weights and architecture. Results reflect prompt-based interaction rather than algorithmic retraining. To improve reproducibility, we provide all prompts, datasets, and statistical scripts in the Supplementary Material. Moreover, these findings are context-specific and cannot be generalized to other forensic scenarios or compared directly with conventional deep-learning architectures, which rely on parameter-level optimization and retraining. Future studies should adopt open-source models and standardized benchmarking protocols.

In conclusion, while generative AI is not yet ready to replace human pathologists, it holds significant promise as a supportive tool in forensic IHC evaluation. Its strengths in speed, consistency, and scalability must be balanced against its limitations in interpretative nuance and legal accountability. With continued development and ethical oversight, AI could become an integral part of the forensic diagnostic landscape.

## 5. Conclusions

This study demonstrates that generative AI, when trained on structured datasets, can effectively support the evaluation of immunohistochemical (IHC) slides in forensic pathology. The AI model achieved high concordance with expert pathologists, particularly in clearly defined staining categories, and showed promising performance in replicating semi-quantitative scoring systems.

The key takeaways from this research are as follows:

- AI can enhance consistency and reduce inter-observer variability in IHC interpretation, addressing one of the major limitations of traditional forensic histopathology.
- Generative models can learn complex visual patterns, offering rapid and reproducible assessments that can streamline forensic workflows.
- Intermediate categories remain challenging, underscoring the need to improve AI sensitivity and interpretative precision.
- AI is not a replacement for human expertise, but a complementary tool that can assist pathologists, especially in high-throughput or resource-limited settings.
- Ethical and legal considerations must guide implementation, ensuring transparency, data protection, and accountability in medico-legal contexts.

By integrating AI into forensic IHC analysis, the field stands to benefit from improved diagnostic precision, operational efficiency, and broader accessibility. However, successful adoption will require continued validation, interdisciplinary collaboration, and a commitment to maintaining the integrity of forensic science.

These results highlight the potential role of AI tools in real forensic contexts. After a rigorous validation process, AI could be used to provide a second, blind evaluation of IHC slides alongside the forensic pathologist. However, it is essential to emphasize that the final diagnostic responsibility must always remain with the pathologist. The use of AI should be strictly limited to assessing the presence or absence of staining (positive/negative), while the evaluation of cell-level immunopositivity remains solely within the pathologist’s expertise. AI is integrated as an adjunct tool, operating after slide digitization and before final reporting. It functions as a second opinion system, reducing variability and accelerating turnaround times while maintaining the pathologist’s authority over the final diagnosis. Future research should prioritize explainable AI and domain-specific CNNs for legally defensible forensic applications.

## Figures and Tables

**Table 1:** Classification criteria for immunoreactivity levels based on cellular staining incidence, following Meyerholz and Beck’s guidelines [24].

Category	Symbol	Cellular Staining Incidence (%)
0	–	No staining (0%)
1	+	Weak staining (1–25%)
2	++	Mild staining (26–50%)
3	+++	Moderate staining (51–75%)
4	++++	Dark staining (>75%)

**Table 2:** Performance of AI during training phase for binary classification (negative vs. positive immunoreactivity). The table reports correct and incorrect evaluations across 150 images (n = 30 negative, n = 120 positive).

Reference Indicator	Incorrect Evaluation	Correct Evaluation
30 “−”	2	28
120 “+”	0	120

**Table 3:** Detailed classification accuracy of AI across five immunoreactivity categories during training (n = 150 images). Columns show error counts, correct evaluations, and percentage accuracy per category.

Category	Reference Indicator	Incorrect Evaluation	Correct Evaluation	Error Percentage	% Correct
“−”	30	2	28	6.67%	93.33%
“+”	30	0	30	0%	100%
“++”	30	0	30	0%	100%
“+++”	30	0	30	0%	100%
“++++”	30	0	30	0%	100%

**Table 4:** Binary classification results (negative vs. positive immunoreactivity) during blind testing phase (n = 75 images). The table summarizes correct and incorrect evaluations.

Reference Indicator	Incorrect Evaluation	Correct Evaluation
15 “−”	1	14
60 “+”	13	47

**Table 5:** Full categorical breakdown of AI classification accuracy during blind testing (n = 75 images). Columns report error counts, correct evaluations, and percentage accuracy per immunoreactivity category.

	Reference Indicator	Incorrect Evaluation	Correct Evaluation	Error Percentage	% Correct
“−“	15	1	14	6.67%	93.33%
“+”	15	4	11	26.67%	73.33%
“++”	15	5	10	33.33%	66.67%
“+++”	15	1	14	6.67%	93.33%
“++++”	15	3	12	20%	80%

**Table 6:** Summary of qualitative observations of AI performance during blind testing. Strengths and weaknesses are categorized based on interpretative behavior and classification patterns.

Aspect	Observation Type
High performance in extreme categories (“−” and “+++”)	Strength
Fast and consistent processing of all images	Strength
No human fatigue or subjectivity	Strength
Clear visual patterns learned effectively	Strength
Lower recall in intermediate categories (“+” and “++”)	Weakness
Confusion between adjacent intensity levels	Weakness
Lack of explanation/interpretability	Weakness
Difficulty with borderline cases	Weakness

## Data Availability

The data presented in this study are available on request from the corresponding author due to legal reasons.

## Supplementary Materials

The following supporting information can be downloaded at: https://www.mdpi.com/article/10.3390/diagnostics16010006/s1, Table S1: Dataset Description; Table S2: Prompt Engineering Workflow.

## Abbreviations

The following abbreviations are used in this manuscript:

AI	Artificial Intelligence
AUC	Area Under the Curve
DL	Deep Learning
IHC	Immunohistochemistry
ML	Machine Learning
ROC	Receiver Operating Characteristic
T0	Training Phase
T1	Blind Evaluation Phase
CNN	Convolutional Neural Network
