# Supplementary material for: AI-Assisted Diagnostic Evaluation of IHC in Forensic Pathology: A Comparative Study with Human Scoring
Source: Diagnostics (Basel). 2025 Dec 19;16(1):6. doi: 10.3390/diagnostics16010006 (PMC12785249; doi:10.3390/diagnostics16010006)
Supplement: Supplementary file 1 [file diagnostics-16-00006-s001.zip › diagnostics-3974196-supplementary.pdf]

Table S1 – Dataset Description

Parameter	Details
Total images	225
Categories	–, +, ++, +++, ++++
Images per category	45
Training set	150 images (30 per category)
Blind testing set	75 images (15 per category)
Image format	TIFF
Magnifications	5×, 10×, 20×, 40×
Acquisition device	Leica microscope
Source	Institute of Forensic Medicine, University of Catania
Inclusion criteria	High-resolution, validated by two forensic pathologists
Exclusion criteria	Poor resolution, technical artifacts, ambiguous staining

Table S2 – Prompt Engineering Workflow

Phase	Prompt Example
Training	"This image has been evaluated as [score]. What is your opinion?"
Blind Testing	"Classify this image according to the following scale: –, +, ++, +++, ++++."
Reinforcement	"Your previous classification was incorrect. The correct score is [score]."

Interaction Notes:

- Prompts were standardized across all images.
- No contextual cues were provided during blind testing.
- All interaction logs are available upon request.

Reproducibility Statement

This study used a proprietary generative AI system (ChatGPT-4V), which limits reproducibility due to lack of access to model weights and architecture. Results reflect prompt-based interaction rather than algorithmic retraining. To improve transparency, all prompts, datasets, and statistical scripts are provided in this Supplementary Material. Future studies should adopt open-source computer vision models (e.g., CNN-based architectures such as ResNet or VGG) and standardized benchmarking protocols to ensure reproducibility and compliance with FAIR principles.
